# Genetic alteration and clinical significance of SUMOylation regulators in multiple cancer types

**DOI:** 10.7150/jca.49042

**Published:** 2020-09-30

**Authors:** Guangzhen Wu, Yingkun Xu, Ningke Ruan, Jianyi Li, Qingyang Lv, Qi Zhang, Yougen Chen, Qifei Wang, Qinghua Xia, Quanlin Li

**Affiliations:** 1 Department of Urology, The First Affiliated Hospital of Dalian Medical University, Dalian, Liaoning, 116011, China.; 2 Department of Urology, Shandong Provincial Hospital, Cheeloo College of Medicine, Shandong University, Jinan, Shandong, 250021, China.; 3 The Nursing College of Zhengzhou University, Zhengzhou, Henan, 450001, China.; 4 Department of Urology, Union Hospital, Tongji Medical College, Huazhong University of Science and Technology, Wuhan, Hubei, 430022, China.; 5 Department of Urology, Shandong Provincial Hospital Affiliated to Shandong First Medical University, Jinan, Shandong, 250021, China.

**Keywords:** SUMOylation, pan-cancer, TCGA, risk signature, overall survival

## Abstract

The purpose of this study was to investigate the genetic variation, gene expression differences, and clinical significance of SUMOylation regulators in pan-cancers. Based on previous studies, we gained a better understanding of the biological process of SUMOylation and the status of current research. In the present study, we employed a wide range of bioinformatics methods. We used genetic variation and mRNA expression data in the Cancer Genome Atlas (TCGA) to construct a panoramic view of the single nucleotide variants, copy number variants, and gene expression changes in SUMOylation regulators in various tumors. Subsequently, we used the String website and the Cytoscape tool to construct the PPI network between these regulators. We used the GSCALite website to determine the relationship between these regulators and cancer pathways and drug sensitivity. We constructed images of co-expression between these regulators using the R programming language. Using clinical data from TCGA, we performed hazard ratio analysis for these regulators in pan-cancer. Most importantly, we used these regulators to successfully establish risk signatures related to patient prognosis in multiple tumors. Finally, in KIRC, we conducted gene-set enrichment analysis (GSEA) of the five molecules in its risk signatures. We found that these five molecules are involved in multiple cancer pathways. In short, we have comprehensively interpreted the detailed biological process of SUMOylation at the genetic level for the first time, successfully constructed multiple risk signatures, and conducted GSEA in KIRC. We believe that these findings provide credible and valuable information that is relevant for future clinical diagnoses and scientific research.

## Introduction

SUMOylation is a process of post-translational modification of a target protein by a small ubiquitin-like modifier (SUMO). The cycle consists of five steps: maturation, activation, conjugation, ligation, and de-modification. Recent research has confirmed that dysregulation of the SUMO system plays an essential role in the occurrence and development of many diseases, especially cancer 1-5 and is widely involved in DNA damage responses, carcinogenesis, cancer cell proliferation, metastasis, and apoptosis 6-9. Since the SUMOylation regulator obviously affects most cellular processes and functions, it is very important in many diseases, including cancer 10-13. It has been suggested that SUMOylation regulators could represent potential cancer treatment targets. Therefore, comprehensive investigation regarding the heterogeneity of SUMOylation regulators in pan-cancers is required.

Extensive sequencing of cancer genomes has already begun, and one of the largest collections of these data can be found in The Cancer Genome Atlas (TCGA). Sequencing efforts have revealed variations in the cancer genome, providing a more comprehensive understanding of cancer biology 14. However, the discovery of a large number of mutant cancer genes has been accompanied by another arduous task: identifying the underlying genes that drive the cancer 15.

In the present study, we systematically explored the gene mutations, mRNA expression, and clinical significance of SUMOylation regulators in various cancer types for the first time. We found that these SUMOylation regulators have a wide range of mutation frequencies and copy number variations in a variety of cancers, and identified that CBX4 may play an oncogenic role in many cancers. In addition, we found that there are complex interactions between SUMOylation regulators, and their activity is closely related to multiple cancer pathways. We then explored the sensitivity between these SUMOylation regulators and anticancer drugs. Subsequently, we explored the relationship between these SUMOylation regulators in pan-cancers and the prognosis of patients. We found that SUMOylation regulators may be a potential indicator of the prognosis of multiple tumors. Finally, we used these SUMOylation regulators to establish risk signatures related to patient prognosis in five cancers (ACC, KIRC, KIRP, LUAD, and SKCM). Because we are committed to renal clear cell carcinoma research, and to explore the potential biological role of these target genes in tumors, we targeted the five SUMOylation regulators (PIAS1, PIAS3, SENP8, SUMO4, and TRIM27) that were present in the risk signature of KIRC upon gene-set enrichment analysis (GSEA) 16.

Our results revealed that SUMOylation regulators are essential for the occurrence and development of various tumors. We believe that our research can provide future researchers with inspiration regarding novel avenues for scientific research and establish a new foundation for clinical diagnosis and treatment.

## Materials and Methods

### Identification of SUMOylation regulators

**Figure 1:**
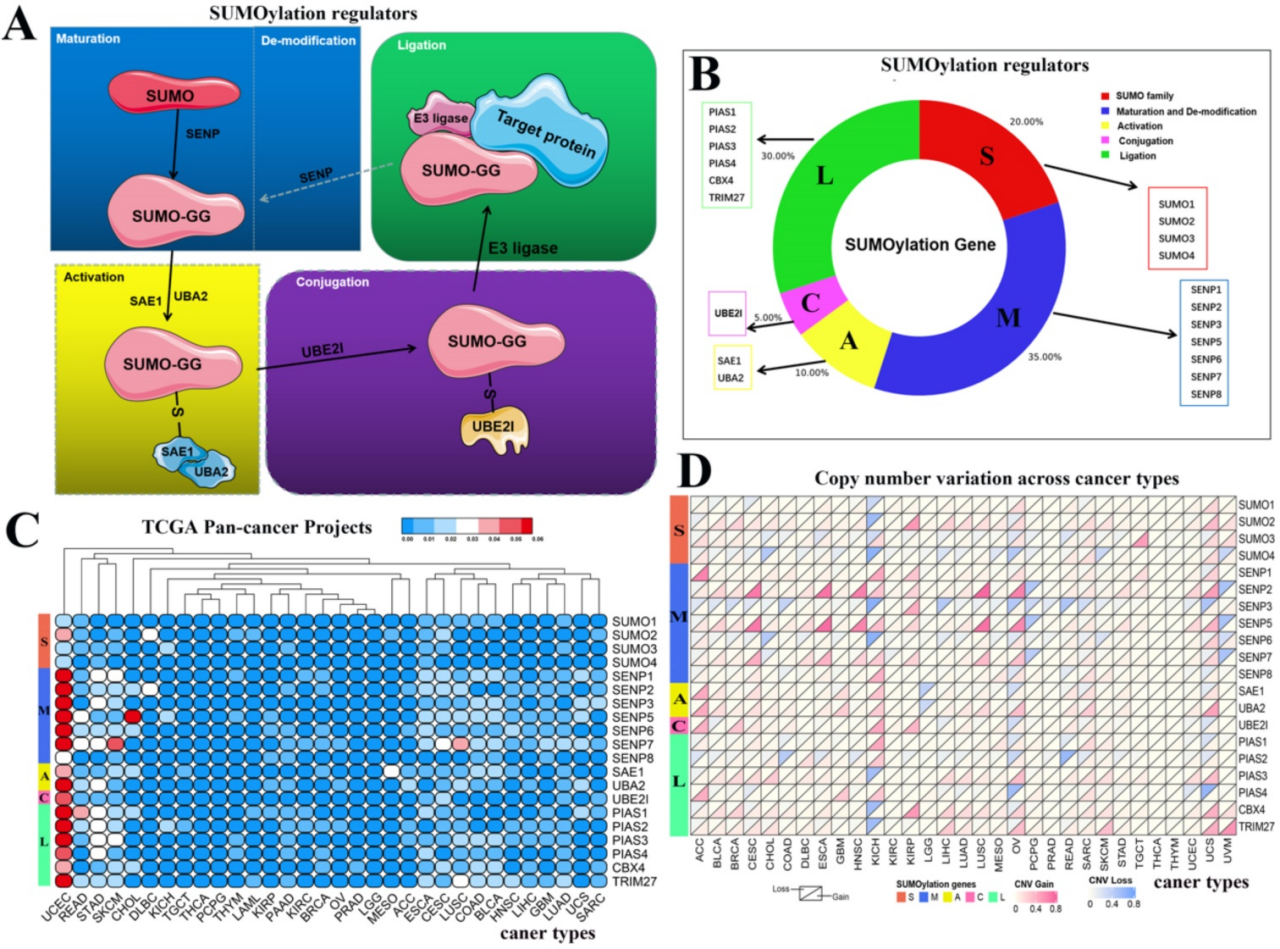
** Schematic diagram of the SUMOylation process and the variation in SUMOylation regulators in pan-cancers.** (**A**) Diagram of SUMOylation regulators. (**B**) Proportions of SUMO family, Maturation and De-modification, Activation, Conjugation, and Ligation groups among SUMOylation regulators. (**C**) Mutation frequency of SUMOylation regulators across 33 cancer types. The redder the color, the higher the degree of variation. (**D**) CNV alteration frequency of SUMOylation regulators across 33 cancer types. The upper part of each grid shows CNV losses, and the bottom part shows CNV gains.

From recently published articles, we identified 20 SUMOylation regulators 4, 17. The SUMO family contains four regulators. Maturation and de-modification contain seven regulators. Activation contains two regulators. Conjugation contains one regulator. Ligation contains six regulators. A schematic diagram of the SUMOylation process is shown. In constructing the schematic diagram of Figure 1A, we use two softwares, Photoshop and PowerPoint. In drawing the image in Figure 1B, we used Photoshop and bioinformatics online tools (http://www.bioinformatics.com.cn/) to complete the image. Bioinformatics.com is a website focused on online data analysis, mapping and visualization.

### Data collection

To better identify, diagnose, and treat tumors, there is an urgent need to conduct in-depth research regarding their genetic changes and establish appropriate databases. In 2006, the National Cancer Institute (NCI) and National Human Genome Research Institute (NHGRI) collaborated on the Cancer Genome Atlas (TCGA) database program. The program aims to identify gene changes caused by tumorigenesis and development through large-scale gene sequencing and comprehensive, multi-dimensional analyses, by building a comprehensive atlas related to tumor genes. The results of our study are based on 'omics' datasets generated by TCGA Research Network (http://cancergenome.nih.gov/). We analyzed 33 different TCGA projects, and each project represented a specific cancer type. We obtained RNA-seq data and clinicopathological data of these 33 types of cancer via this database. Taking KIRC as an example, the KIRC dataset in TCGA database includes 72 normal samples and 539 renal clear cell carcinoma samples.

### GEPIA dataset

GEPIA (http://gepia.cancer-pku.cn/) is based on TCGA database and used to analyze the RNA sequence expression data of more than 9,000 tumors and 8,000 tumor genome maps 18. We explored the expression of the *CBX4* gene in a variety of cancers through this online database, and *P* < 0.05 was considered statistically significant.

### Generation of PPI networks

STRING is a high-coverage, high-quality protein-protein interaction network platform with a wide range of applications for interpreting biomedical 'big data' and visualization in the context of systems biology 19. We used the STRING online database to map SUMOylation regulators in the PPI network. We then used the visualization software Cytoscape to embellish the PPI network 20. The data in PPI were used to construct a quantization table.

### GSCALite analysis

GSCALite (http://bioinfo.life.hust.edu.cn/web/GSCALite/) is an interactive web-based web application for genomic cancer analysis that analyzes and visualizes genome expression/variation/correlation in cancer in a flexible way 21. The report provided by GSCALite includes gene differential expression, overall survival, single nucleotide variation, copy number variation, methylation, pathway activity, miRNA regulation, normal tissue expression, and drug sensitivity. We used this online tool to explore the relationship between SUMOylation regulators and cancer pathways, and finally to investigate their sensitivity to multiple anticancer drugs.

### Bioinformatics analysis and data processing

In our research, we used bioinformatics to achieve our research goals. We made extensive use of the Perl and R programming languages to analyze and visualize data. We explored the single nucleotide variants (SNVs), copy number variants (CNVs), and gene expression variations of SUMOylation regulators in various tumors via the TCGA database. We used the Limma software package for differential analysis. TBtools was used for heat map post-processing. To explore the expression relationship between these regulators, we plotted co-expression maps of SUMOylation regulators in ACC, BLCA, PRAD, and KIRP tumors. We used the Corrplot software package to complete the co-expression analysis. To explore whether these regulators are risk or protective factors in pan-cancers, we performed a hazard ratio analysis on the SUMOylation regulators in pan-cancers. Our primary research area is renal clear cell carcinoma, and we displayed the hazard ratio results of these SUMOylation regulators in KIRC in the form of a forest chart. In addition, we observed that PIAS3 might play a more critical role in a variety of tumors, so we displayed the hazard ratio results of PIAS3 in several tumors in the form of a forest chart. We then used these SUMOylation regulators to establish risk signatures related to patient prognosis in five types of tumors, including ACC, KIRC, KIRP, LUAD, and SKCM. We plotted survival curves, ROC curves, and multivariate independent prognostic analyses. The Pheatmap software package was used to construct heat maps. The survival software package was used to analyze and construct survival curves. The SurvivalROC software package was used to explain and illustrate the ROC curve. Finally, we conducted GSEA in KIRC for five molecules: PIAS1, PIAS3, SENP8, SUMO4, and TRIM27. *P* < 0.05 was considered statistically significant.

## Results

### Variation and expression of SUMOylation regulators in various cancers

SUMOylation has been widely studied by researchers, and the SUMOylation regulators involved in this process have been identified. The cycle consists of five steps: maturation, activation, conjugation, ligation, and de-modification (Fig. 1A). These regulators can be divided into five parts: SUMO family, maturation and de-modification, activation, conjugation, and ligation. We constructed a composition diagram for these five parts (Fig. 1B). The SUMO family contains four regulators. Maturation and de-modification include seven regulators. Activation contains two regulators. Conjugation contains one regulator. Ligation provides six regulators. Subsequently, we explored the mutation frequency and copy number variation frequency in SUMOylation regulators in 33 cancer types via TCGA Pan-Cancer Project (Fig. 1C, 1D). These SUMOylation regulators have lower overall average mutation frequencies in 33 types of cancer, although SENP1, SENP5, SENP7, and PIAS3 regulators have higher mutation frequencies. We found a higher overall mutation frequency in both UCEC and STAD cancers (Additional file 1: Table S1).

**Figure 2:**
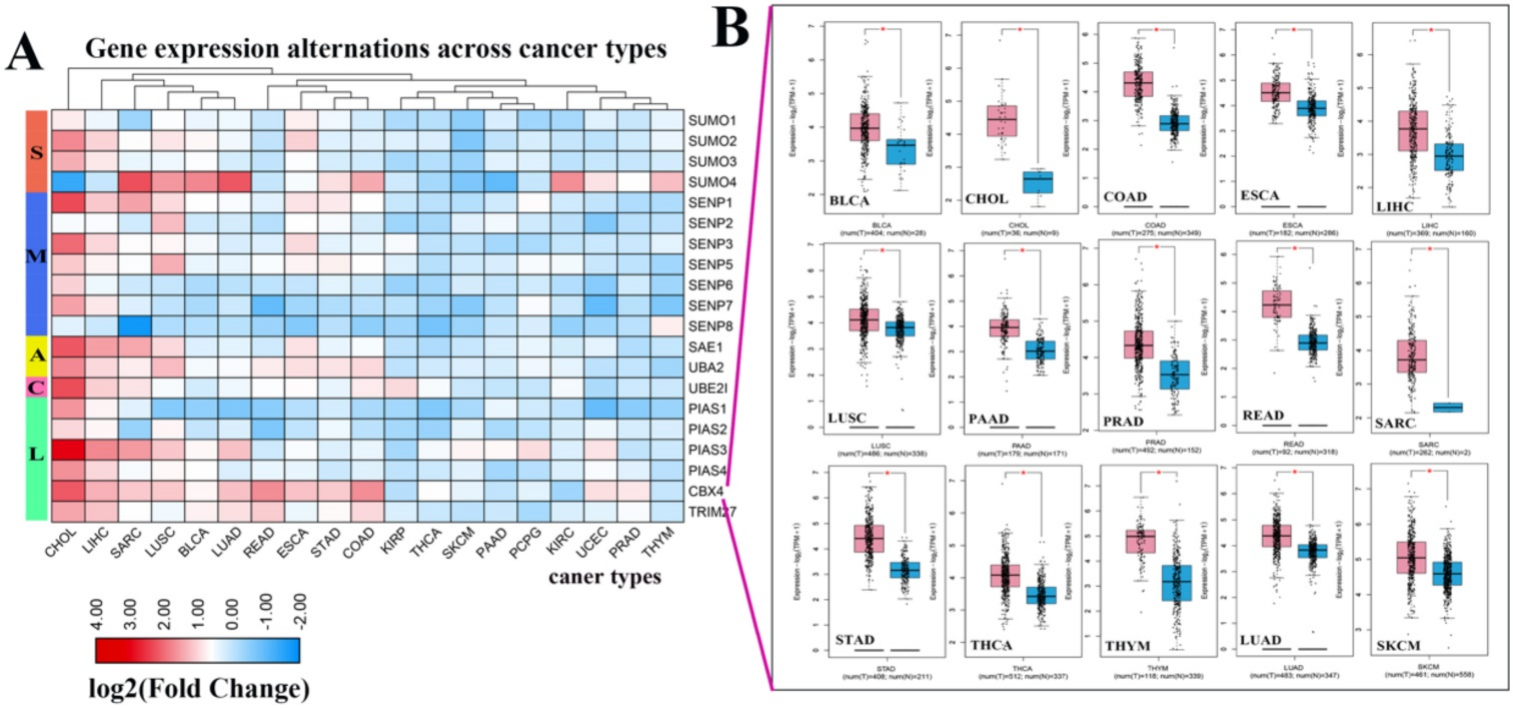
** Expression levels of SUMOylation regulators in various tumors.** (**A**) Gene expression alterations of SUMOylation regulators across 19 cancer types. The heat map shows the fold change, with blue representing down-regulated genes, and red representing up-regulated genes. (**B**) Box plots showing the expression of CBX4 between 15 types of cancer samples and their corresponding normal samples. Red represents tumor tissue and blue represents normal tissue. *P < 0.05.

Next, we investigated the frequency of CNVs in these SUMOylation regulators in 33 types of tumors and found that CNV changes are ubiquitous. Among them, SENP2, SENP5, CBX4, and TRIM27 showed more extensive CNV amplification; in contrast, SENP3 and SUMO4 had more extensive CNV deletions. After careful comparison of the mutations between various cancers, we found that a wide range of CNV gains are seen in KICH and UCS cancers, and a wide range of CNV losses are seen in KICH and OV cancers (Additional file 1: Table S2, S3). To investigate whether these genetic variations affect the expression of SUMOylation regulators, we explored the expression perturbations of SUMOylation regulators in 19 cancer types. We used the R language to construct a heat map of the expression alterations of these regulators across multiple tumors and graphically enhanced them using TBtools (Fig. 2A). Compared with healthy tissues, CBX4, a regulator of SUMOylation with extensive CNV amplification, showed significantly higher expression in cancer tissues (Fig. 2B). Past research has demonstrated that CBX4 is involved in DNA damage and repair processes 22.

In contrast, PIAS4, a regulator with extensive CNV deletions, showed significantly lower expression in cancer tissues (Additional file 1: Table S4). Therefore, we speculate that there is a correlation between CNV and gene expression alterations. These results show the genetic variation and expression changes of SUMOylation regulators across multiple types of cancer, suggesting that SUMOylation regulators may play an essential role in many types of cancer.

### The interaction between SUMOylation regulators, their interaction with cancer pathways, and their sensitivity to anticancer drugs

**Figure 3:**
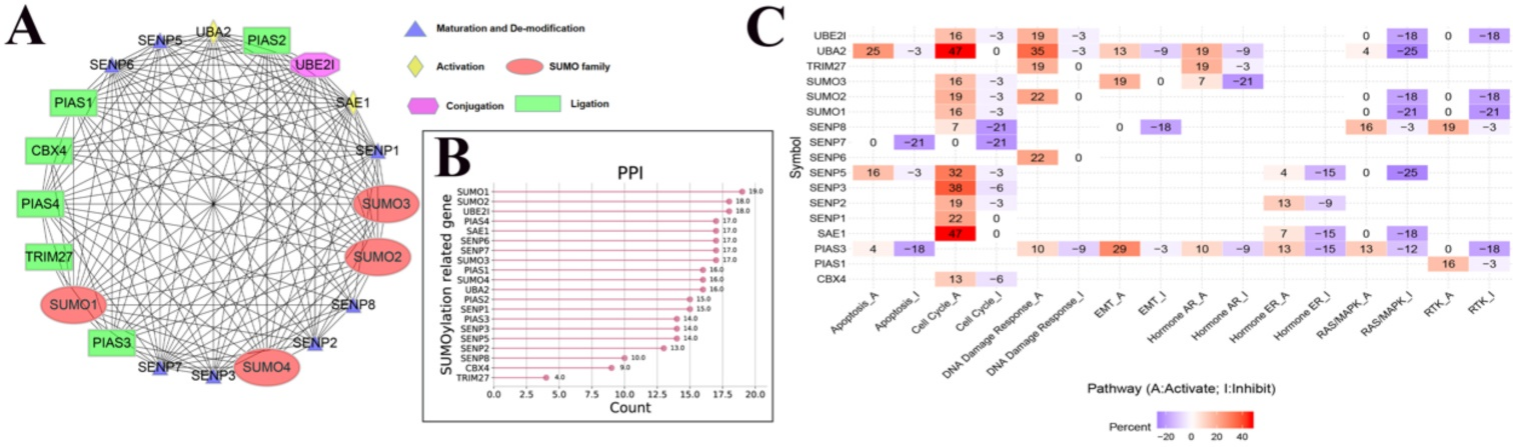
**The PPI network between SUMOylation regulators and the relationship with cancer pathways.** (**A**) Protein-protein interactions among SUMOylation regulators. (**B**) Quantitative maps of PPI were generated between SUMOylation related genes. (**C**) Relationship between SUMOylation regulators and cancer pathways. Positive numbers represent activation and negative numbers represent inhibition. The higher the absolute value, the stronger the correlation with this cancer pathway.

To further understand the potential mechanism of action of SUMOylation regulators in various cancers, we first analyzed the regulatory relationships between each SUMOylation regulator using the STRING website online tool, visualized these using Cytoscape software (Fig. 3A), and finally quantified their relationship (Fig. 3B). It is apparent that SUMO1, SUMO2, and UBE2I play more prominent roles. Subsequently, using the GSEALite website, the relationship between regulators and the activities of various cancer pathways was explored. We found that UBA2, SENP3, and SAE1 are related to activation of the cell cycle pathway, and UBA2, SUMO1, and SENP5 are related to inhibition of the RAS/MAPK pathway (Fig. 3C).

**Figure 4:**
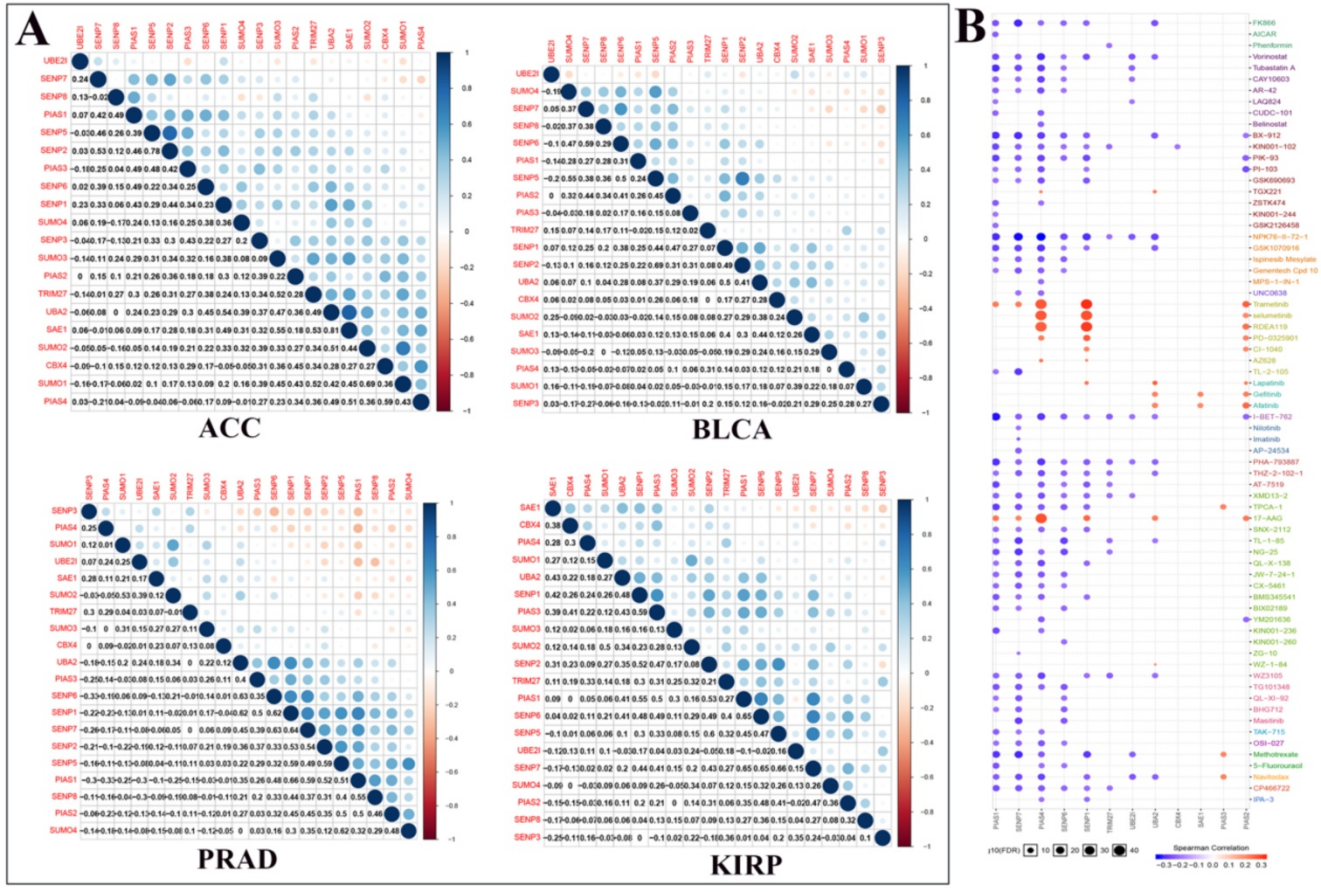
** Co-expression of SUMOylation regulators in four tumors and drug sensitivity analysis.** (**A**) Co-expression of SUMOylation regulators in ACC, BLCA, PRAD, and KIRP. The upper part of the picture shows that the circles between the two molecules are closer to blue in color, indicating that the positive correlation between them is more reliable; the circles between the two units are closer to red in color, meaning that the negative relationship between them is more reliable. The lower part of the picture shows the quantitative value of the relationship between the two molecules. The closer the value is to 1, the stronger the positive correlation; the closer the value is to -1, the stronger the negative correlation. (**B**) Schematic diagram of the sensitivity between the SUMOylation regulators and various anticancer drugs. The color represents the Spearman correlation, and the size represents the strength of drug targeting.

We performed correlation analysis between two regulators in ACC, BLCA, PRAD, and KIRP cancers (Fig. 4A). For example, in ACC, there is a clear positive correlation between UBA2 and SAE1. In BLCA, there is a clear positive correlation between SENP5 and SENP2. In PRAD, there is a clear negative correlation between PIAS4 and PIAS1. In KIRP, there is a significant negative correlation between SAE1 and SENP3. We used Genomics of Drug Sensitivity in Cancer data to analyze the sensitivity of these regulators to anticancer drugs (Fig. 4B). The color represents the Spearman correlation, and the size represents the strength of drug targeting. These results indicate that the SUMOylation regulators play a variety of roles in the occurrence and development of different types of cancer.

### Clinical correlation between SUMOylation regulators and pan-cancer types and establishment risk signatures in multiple tumors

**Figure 5:**
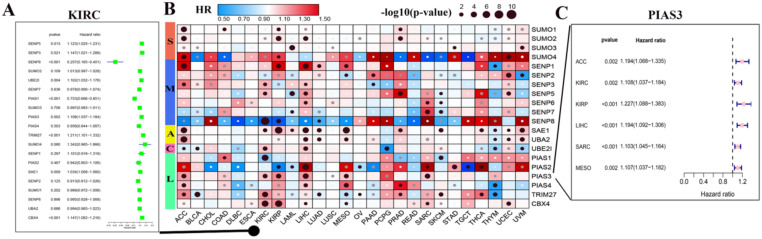
** Hazard ratio analysis of SUMOylation regulators in pan-cancers.** (**A**) Hazard ratios of the SUMOylation regulators in KIRC. (**B**) Hazard ratios of SUMOylation regulators in pan-cancers. Red represents risk factors and blue represents protective factors. The larger the circle, the smaller the *P*-value and the more statistically significant. (**C**) Hazard ratios of PIAS4 in various tumors.

Through the above analysis of SUMOylation regulators across cancer types, we can conclude that there is a wide range of genetic variations and expression changes, which may provide valuable insights for clinical diagnosis and treatment. First, we explored whether these SUMOylation regulators are significantly associated with overall survival in patients with 33 types of cancer (Fig. 5B; Additional file 1: Table S5, S6). We then performed univariate Cox regression analyses of each regulator in KIRC (Fig. 5A). In addition, PIAS3 plays a risk factor in six cancers, ACC, KIRC, KIRP, LIHC, SARA, and MESO (Fig. 5C). These observations suggest that PIAS3 may function as an oncogene in multiple cancer types.

**Figure 6:**
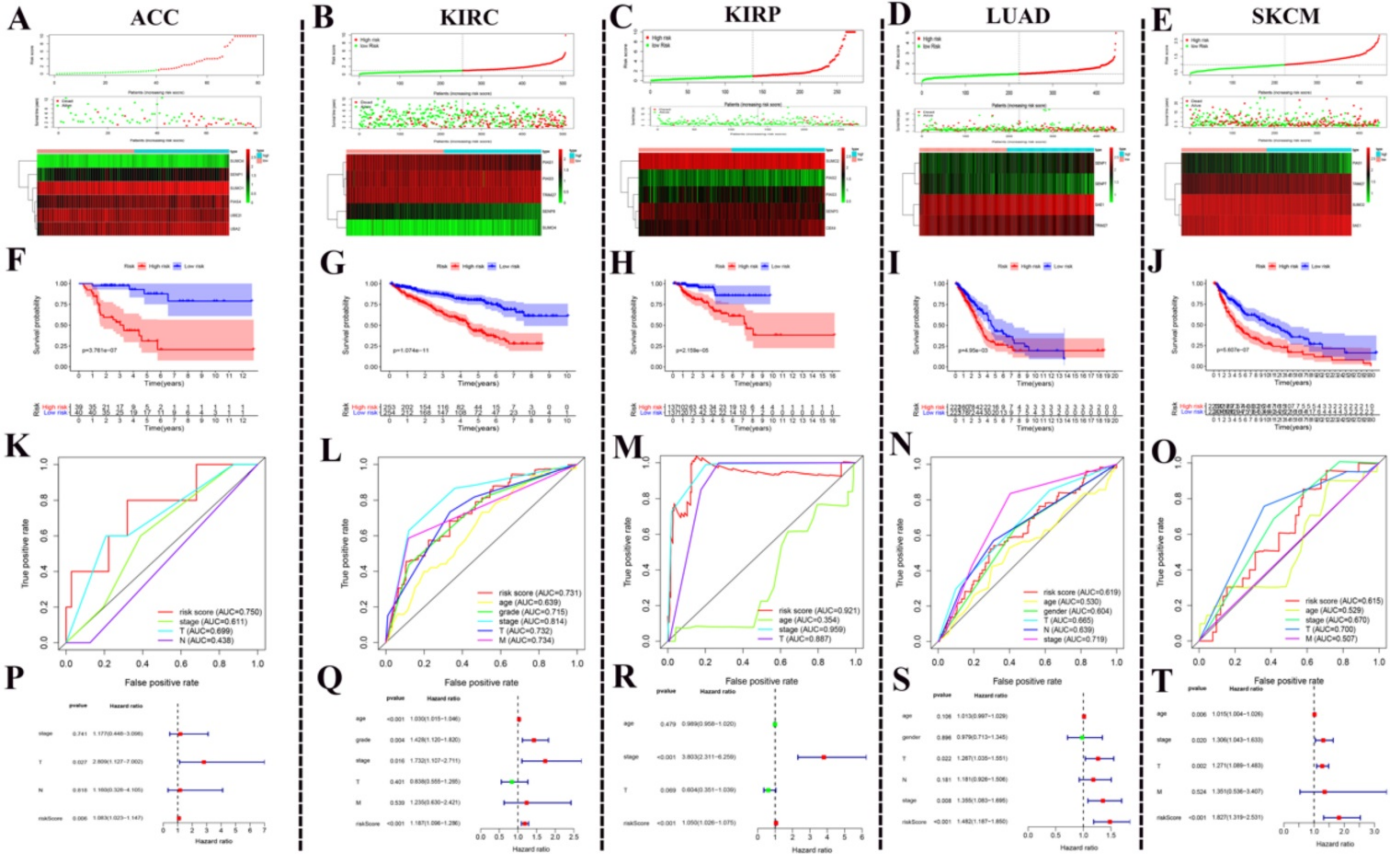
** Using SUMOylation regulators to establish risk signatures in five tumors.** (**A-E**) These regulators were used to create risk signatures in five cancers: ACC, KIRC, KIRP, LUAD, and SKCM. (**F-J**) Based on the corresponding risk signature, a corresponding survival curve was plotted. (**K-O**) Based on the relevant risk signature, the corresponding ROC curve was plotted. (**P-T**) Based on the related risk signature, the corresponding multivariate independent prognostic analysis is drawn.

After the above investigation, we wondered whether the different expression of these regulators could be used to divide cancer patients into groups with different prognoses. We then validated our ideas in five cancers: ACC, KIRC, KIRP, LUAD, and SKCM. We first determined which procedure to use to build this risk signature. In ACC, this risk signature is composed of six genes: SUMO4, SENP1, SUMO1, PIAS4, and UBE2I (Fig. 6A). In KIRC, this risk signature is composed of five genes: PIAS1, PIAS3, TRIM27, SENP8, and SUMO4 (Fig. 6B). In KIRP, this risk signature is composed of five genes: SUMO2, PIAS2, PIAS3, SENP3, and CBX4 (Fig. 6C). In LUAD, this risk signature is composed of four genes: SENP1, SENP7, SAE1, and TRIM27 (Fig. 6D). In SKCM, this risk signature is composed of four genes: PIAS1, TRIM27, SUMO2, and SAE1 (Fig. 6E). According to this risk signature, patients were divided into a high-risk group and a low-risk group, and the overall survival curve was plotted. We were surprised to find a clear prognostic difference between our high and low-risk groups (Fig. 6F-J). We then plotted the ROC curve to verify the accuracy of its risk signature (Fig. 6K-O). Finally, we performed multivariate Cox regression analyses to test which factors were independent risk factors (Fig. 6P-T). In summary, the above results indicate that SUMOylation regulators can play a prognostic stratification role in some types of cancer and have excellent potential as new targets for clinical treatments in the future.

### Results of GSEA in KIRC

**Figure 7:**
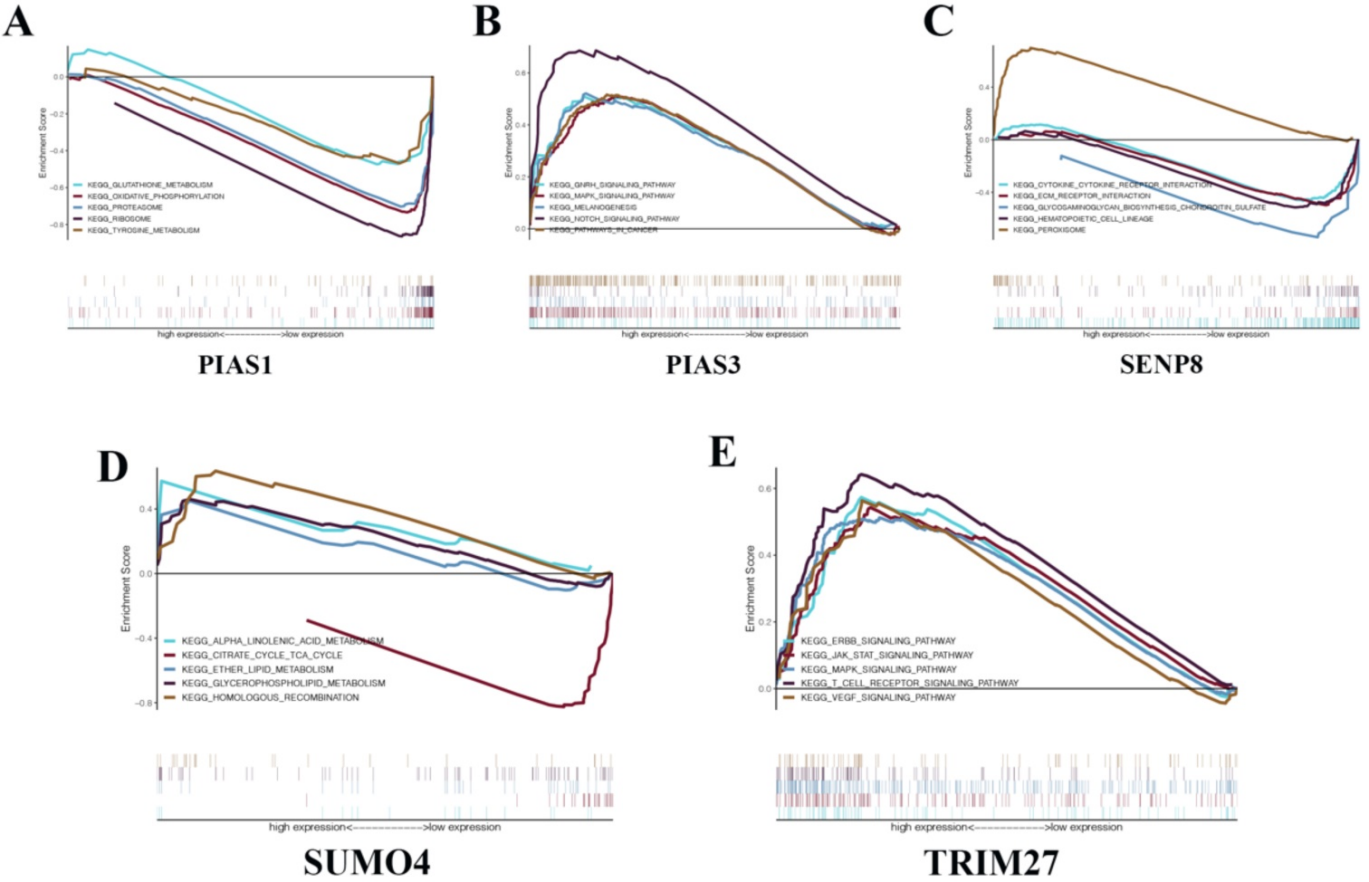
** GSEA in KIRC.** (**A**) PIAS1. (**B**) PIAS3. (**C**) SENP8. (**D**) SUMO4. (**E**) TRIM27.

To gain a deeper understanding of the biological functions of the five genes that make up the KIRC risk signature, we conducted GSEA. We found that PIAS1 can inhibit glutathione metabolism, oxidative phosphorylation, proteasome, ribosome, and tyrosine metabolism biological pathways (Fig. 7A). PIAS3 can activate the GnRH signaling pathway, MAPK signaling pathway, melanogenesis, NOTCH signaling pathway, and biological cancer pathways (Fig. 7B). SENP8 can inhibit cytokine-cytokine receptor interaction, Ecm receptor interaction, glycosaminoglycan biosynthesis chondroitin sulfate, and hematopoietic cell lineage biological pathways. SENP8 can activate the peroxisome biological pathway (Fig. 7C). SUMO4 can activate alpha-linolenic acid metabolism, ether lipid metabolism, glycerophospholipid metabolism, and homologous recombination biological pathways. SUMO4 can inhibit the citrate cycle TCA cycle biological pathway (Fig. 7D). TRIM27 can activate the ERBB signaling pathway, JAK-STAT signaling pathway, MAPK signaling pathway, T cell receptor signaling pathway, and VEGF signaling pathway (Fig. 7E).

## Discussion

Globally, cancer is the leading cause of death in humans. Moreover, as the population grows, age increases, and cancer-related adverse risk behaviors increase, it is expected that the number of new cases of cancer or deaths will increase rapidly. Harmful risk behaviors related to cancer include the use of large amounts of tobacco, lack of adequate exercise, and body weight above the normal range 23. Here, we use the United States as an example. In 2019, the American Cancer Society predicted that more than 1.7 million new cancer cases and 600,000 cancer deaths occurred in the United States 24. Cancer research has always been a popular research area for scientific researchers worldwide.

The biological process of SUMOylation is a fundamental post-translational modification process in the human body that can regulate almost all cellular physiological functions and pathological processes. For example, the control of gene expression levels, regulation of transcription processes, nucleocytoplasmic transport operations, remodeling of chromatin processes, biological signaling processes, mRNA maturation processes, cell division processes, and regulation of the cell cycle and cell proliferation 6, 25-29. In cancer research, researchers have found that SUMOylation plays an increasingly important role in the occurrence and development of human tumors. In the SUMO signaling pathway, changes in the expression or activity of the constituent molecules can completely alter the traits of the cell. Therefore, the SUMO biological pathway can induce cell proliferation, apoptosis, and metastasis by regulating disease-related units.

Previous studies have shown that SENP1 is highly expressed in human prostate cancer specimens and is correlated with the expression of hypoxia-inducible factor 1α (HIF1α). SENP1 induces the transformation of healthy prostate into precancerous lesions *in vitro* and *in vivo* 30. Besides, PIAS1 and PIAS4 are required during the repair process after DNA damage 31. In hematological malignant proliferative diseases, SUMO1 can stimulate the proliferation of leukemia cell lines by regulating IGF-1R 32. In hematological malignant proliferative diseases, SUMO1 can stimulate the proliferation of leukemia cell lines by regulating IGF-1R 32. In digestive system tumors, some researchers have found a correlation between the expression of SAE2 and the expression of C-MYC in gastric cancer tissues. When the expression level of SAE2 was suppressed, the growth and proliferation of gastric cancer cell lines were inhibited 33. Silencing the *SUMO1* gene can inhibit the proliferation of gastric cancer cell lines and promote apoptosis 34. In a subcutaneous tumor-bearing model of nude mice, SENP1 can enhance the invasion and lung metastasis of triple-negative breast cancer cell lines 34. SENP1 can regulate MMP-2 and MMP-9 through the HIF1α signaling pathway, thereby promoting the progression of prostate cancer cell lines and bone metastasis 35. Besides, SENP2 can inhibit the invasion and metastasis of bladder cancer cell lines by affecting the expression of MMP-13 36. Collectively, these data motivated us to carry out in-depth exploration in pan-cancers, centering on SUMOylation-related molecules.

In our study, we observed that CBX4 is highly expressed in various tumors. Previous studies have shown that CBX4 can promote tumor progression by inducing VEGF expression and angiogenesis in hepatocellular carcinoma 37. In *in vitro* animal experiments, CBX4 was able to promote the progression and metastasis of nude mice orthotopic tumor transplantation 38. CBX4 is suggested to play an oncogenic role in various tumors. In addition, in exploring the relationship between these regulators and cancer pathways, we found that most of these regulators have a significant activation effect in the cell cycle pathway, and most of them in the RAS/MAPK pathway have a significant inhibitory effect. The biological effects of these regulators in tumorigenesis and development are diverse. Subsequently, the results of the investigation into the sensitivity of these regulators to mainstream anticancer drugs can assist the formulation of future personalized treatment and precision medicine. In the hazard ratio analysis of these regulators in pan-cancers, we found many meaningful results. Since we have been focusing on the research of clear cell carcinoma of the kidney for a long time 39, we separately showed the results of the hazard ratio analysis of these regulators in KIRC. We can see that SENP5, SENP3, UBE2I, PIAS3, TRIM27, SAE1, and CBX4 are risk factors in the development of renal cell carcinoma. SENP8 and PIAS1 play protective roles in renal clear cell carcinoma. These are all worthy of future attention. In addition, we used these regulators to establish risk signatures related to the prognosis of corresponding tumor patients in multiple tumors. These five risk signatures are independent risk factors for the corresponding tumor in multivariate analysis.

The results of ROC curve analysis indicate that these risk signatures have good prediction accuracy for the corresponding tumors. Particularly in ACC, the prediction accuracy of the relevant risk model is obviously due to other clinical indicators. We believe that these models can play an essential role in future clinical diagnosis and treatment. The risk signature of KIRC consists of five molecules: PIAS1, PIAS3, TRIM27, SENP8, and SUMO4. To explore the possible biological pathways of these molecules in KIRC, we conducted GSEA in KIRC for each unit. We found that although these molecules have been studied in many other tumors, research in KIRC has rarely been carried out. In the future, we will conduct more in-depth research with respect to these molecules in KIRC. We believe that the results of these GSEA analyses can provide potential new areas for future scientific research.

There is an increasing amount of research surrounding SUMOylation, and the main focus has been on inhibitors of SUMOylation regulators 40. Some researchers have used corresponding inhibitors to inhibit the expression of SAE and SENP1/2, leading to the phenomenon of blocking protein maturation or activation, thereafter downstream biological events will be inhibited, which is useful in cancer treatment and has high application potential 41-44. Inhibitors of SUMO E1 ligase have the advantages of higher selectivity and fewer side effects 45. However, current research should not only focus on SUMOylation regulator inhibitors, but also should be more focused on SUMOylation regulator activators, such as cysteine protease polypeptides. Studies have shown that cysteine protease polypeptides can act as SENP analogs and have the ability to cleave SUMO from the target protein and/or cleave the SUMO precursor form to release its active form. SENP analogs can be used to treat various cancers 46. In general, the application of inhibitors and agonists of SUMOylation regulators in the treatment of cancer is crucial. Therefore, future research should focus on determining the role of inhibitors and agonists of SUMOylation regulators in different cancers. We believe that more research is needed in the near future to understand the potential mechanism of these SUMOylation regulators in the process of tumor development, predict the overall survival of cancer patients, and provide a variety of options to combat cancer progression.

## Conclusions

In this study, we first explored the genetic variation, expression differences, and clinical relevance of SUMOylation regulators in various cancers. We demonstrated the co-expression relationship between SUMOylation regulators and the connection with cancer pathway activity. In addition, we used these SUMOylation regulators to establish risk models related to patient prognosis in ACC, KIRC, KIRP, LUAD, and SKCM. Finally, we focused on KIRC and conducted GSEA of the five molecules in its risk signature, showing that these key molecules are related to multiple cancer pathways. In conclusion, our results reveal the potential mechanism of action of SUMOylation regulators in cancer, provide valuable suggestions for future areas of scientific research, and highlight potential therapeutic targets for future clinical treatment options.

## Supplementary Material

Supplementary tables.

## Abbreviations

TCGA: The Cancer Genome Atlas
CBX4: Chromobox 4
SNV: Single nucleotide variant
CNV: copy number variation
UCEC: uterine corpus endometrial carcinoma
STAD: stomach adenocarcinoma
KICH: kidney chromophobe
UCS: uterine carcinosarcoma
OV: ovarian serous cystadenocarcinoma
PPI: protein-protein interaction
ACC: adrenocortical carcinoma
BLCA: bladder urothelial carcinoma
PRAD: prostate adenocarcinoma
KIRP: kidney renal papillary cell carcinoma
KIRC: kidney renal clear cell carcinoma
LUAD: lung adenocarcinoma
SKCM: skin cutaneous melanoma
SENP: SUMO specific peptidase
PIAS: protein inhibitor of activated STAT
IGF-1R: insulin-like growth factor 1 receptor
C-MYC: MYC proto-oncogene, BHLH transcription factor
SAE: SUMO1 activating enzyme
MMP: matrix metallopeptidase
SUMO: small ubiquitin like modifier
UBE2I: ubiquitin conjugating enzyme E2 I
VEGF: vascular endothelial growth factor
TRIM27: tripartite motif containing 27
GSEA: gene set enrichment analysis
